# Miss Nightingale and the City of London

**Published:** 1908-03-21

**Authors:** 


### MISS NIGHTINGALE AND THE CITY OF LONDON.

On March 16, in the Council Chamber of the Guildhall, was carried out the ceremony of presenting the resolution offering the honorary Freedom of the City to Miss Florence Nightingale, O.M., represented by her relative Mr. L. H. S. Nightingale. There was a large and appreciative audience, both on the floor of the chamber and in the galleries: a number of girls from City schools were allotted places in the latter, where also two ladies of the Army Nursing Service were conspicuous in their brilliant uniforms. On the arrival of the Lord Mayor and retinue proceedings began with the reading by the Town Clerk of the order of the Court of Common Council. The City Chamberlain, Sir Joseph Dimsdale, then delivered an address, setting forth the services to humanity and the nation for which the Corporation had decided to offer the Freedom of the City. His reference to the unexplained omission of previous generations, and to the regret of all that the advanced age and failing health of the "free sister" precluded her personal attendance, provoked sympathetic murmurs.

After accepting the oak casket in which, by Miss Nightingale's special request, the resolution was enclosed together with a cheque for charities representing the value of the usual gold one, Mr. L. H. Shore Nightingale made a brief and dignified reply on behalf of his relative, claiming that whatever Florence Nightingale did was done for no personal end or motive, but from her interest in the work and her conviction that what was worth doing at all was worthy of her best efforts.

Mr. H. Bonham Carter, also a relative of the heroine o the day, then addressed the meeting. Having been ass ciated with Miss Nightingale in childhood, and with h later work as Secretary of the Nightingale Fund for fort years, Mr. Bonham Carter was content to dwell less upo the Crimean period, and to supply personal reminiscenc of his august relative and her work in this country.

Mr. Deputy Wallace then moved that the address of t Chamberlain with Mr. Nightingale's answer should entered in the Journal of the Court and printed in t minutes. This was carried unanimously, and the procee ings terminated.

The presentation souvenir distributed in the Coun Chamber set out the following as the particulars of Mi Nightingale's work and career:—

Nursing and Organisation in the Crimea and at Scuta: 1854, 1855, 1856.
National Testimonial, known as the "Nightingale Fund 1856.
Royal Commission on the Sanitary State of the Army, 185
Female Nursing and Organisation in the British Arm 1858.
Sanitary Conditions of Hospitals and Hospital Constru tion, 1858.
"Notes on Nursing," published about 1859.
St. Thomas's Hospital Training School for Nurses esta lished by the Nightingale Fund, 1860.
Sanitary State of the Army in India, 1863.
District Nursing and Workhouse Infirmaries, 1876.

